# Indian Delphi consensus on cardiovascular risk mitigation in prostate cancer management

**DOI:** 10.3389/fonc.2026.1787811

**Published:** 2026-07-16

**Authors:** Ganesh Bakshi, Alexander Lyon, Amit Joshi, Vivek Agarwala, S. K. Raghunath, Sanjai Kumar Addla, Rajesh Taneja, Anil Mandani, Bhalchandra Kashyapi, Saurabh Bhargava, Rakesh Sharma, Vinayak Agrawal, Ravi Nagar, R. Srivathsan, Keval Patel, Raj Patel, Hetan Shah, Allen Lai, Apurba Mukherjee, Sudhanshu Pandey, Aashishsingh Rajput

**Affiliations:** 1 Uro-oncology, P D Hinduja Hospital and Medical Research Center, Mumbai, Maharashtra, India; 2 Royal Brompton Hospital, London, United Kingdom; 3 Dept. Of Medical Oncology, Dept. Of Medical Oncology, Tata Memorial Hospital, Navi Mumbai, Maharashtra, India; 4 Medical Oncology & Hemat Oncology, & Consultant Cardio Oncologist, Narayana Health, Howrah/Kolkata, West Bengal, India; 5 HCG Bangalore Institute of Oncology, Bangalore, Karnataka, India; 6 Apollo Cancer Institute, Hyderabad, Telangana, India; 7 Andrology and Robotic Surgery, Indraprastha Apollo Hospitals, New Delhi, India; 8 Urology and Kidney Transplant, Medanta The Medicity, Gurugram, Delhi, India; 9 Deenanath Mangeshkar Hospital, Pune, Maharashtra, India; 10 Mazumdar Shaw Medical Centre, Bangalore, Karnataka, India; 11 Basavatarakam Indo-American Cancer Hospital, Hyderabad, Telangana, India; 12 Fortis Memorial Research Institute (FMRI), Gurugram, Delhi, India; 13 Kokilaben Dhirubhai Ambani Hospital, Indore, Madhya Pradesh, India; 14 Apollo Hospitals, Chennai, Tamil Nadu, India; 15 Aayushyam Speciality Hospital, Ahmedabad, Gujarat, India; 16 Zydus Cancer Hospital, Ahmedabad, Gujarat, India; 17 Department of Cardiology, Sheth Gordhandas Sunderdas (GS) Medical College and King Edward Memorial (KEM) Hospital, Mumbai, Maharashtra, India; 18 Ferring Pharmaceuticals, Singapore; 19 Japan, Australia, Korea (JAK), Southeast Asia (SEA) & India, Medical Operations, Ferring Pharmaceuticals, Singapore; 20 Ferring Pharmaceuticals, Mumbai, Maharashtra, India

**Keywords:** androgen deprivation therapy (ADT), androgen receptor pathway inhibitors (ARPIs), cardio-oncology, cardiovascular risk, Delphi method, India, multidisciplinary management, prostate cancer

## Abstract

**Introduction:**

Prostate cancer (PCa) is one of the most common cancers affecting men worldwide and its prevalence is rising in India. The disease primarily affects older men, with incidence increasing with age, and a substantial proportion present with advanced or metastatic disease at diagnosis. Simultaneously, cardiovascular disease (CVD) remains highly prevalent in the Indian population, representing a major cause of non-cancer mortality. The intersection of PCa, its treatment, and cardiovascular comorbidities presents a complex clinical challenge, particularly as therapies such as androgen deprivation therapy (ADT) and androgen receptor pathway inhibitors (ARPIs) improve overall survival but carry cardiometabolic risks.

**Methods:**

The Prostate Cancer Cardiovascular (PCCV) Risk Expert Panel, consisting of urologists, oncologists, and cardio-oncologists from India with guidance from an international expert, developed practical, India-specific recommendations using a modified Delphi methodology. Evidence on the cardiovascular effects of ADT and ARPIs was reviewed and contextualized for Indian clinical practice. Consensus was defined as ≥75% agreement among panelists.

**Results:**

The panel recommended comprehensive baseline lifestyle and cardiometabolic assessment, use of simplified cardiovascular risk checklists, baseline ECG and metabolic profiling, and risk stratification into low, intermediate, or high cardiovascular risk. GnRH antagonists were preferred for high-risk patients, with referral to cardiologists for those at intermediate or high risk. Ongoing cardiometabolic monitoring, lifestyle interventions, and integration of the ABCDE (A – Awareness and Aspirin: Identify risk factors and consider prophylactic aspirin if appropriate, B – Blood Pressure: Monitor and manage hypertension, C – Cholesterol/Cigarettes: Manage cholesterol and avoid smoking, D – Diet/Diabetes: Promote a heart-healthy diet and control blood sugar, E – Exercise: Encourage regular physical activity). Cardio-Oncology framework were emphasized. A multidisciplinary care model involving oncologists, urologists, cardiologists, and primary care providers was strongly recommended.

**Conclusion:**

With increasing use of ADT and ARPIs and the high burden of cardiovascular comorbidities in Indian men with PCa, proactive cardiovascular risk assessment, individualized treatment selection, and coordinated multidisciplinary management are essential. This expert consensus provides actionable, India-specific guidance to balance oncologic efficacy with cardiovascular safety and improve long-term outcomes and quality of life for patients.

## 1 Introduction

Prostate cancer (PCa) is among the most common cancers affecting men globally and is increasingly prevalent in India. In 2020, PCa was among the top three most frequently diagnosed cancers in men across 20 of 47 Asian countries, accounting for almost one-third of global PCa-related deaths (1). In India, prostate cancer incidence but is steadily increasing due to longer life expectancy, lifestyle changes, and improved healthcare access. It predominantly affects older men, with a mean age at diagnosis of around 71 years and incidence increases sharply after 50 years, with the highest rates observed in urban regions (2).

A large proportion of Indian patients present with advanced or metastatic disease (43–77%), indicating delayed diagnosis and limited awareness (3, 4). Furthermore, PCa patients in India face a heightened risk of cardiovascular mortality due to advanced age, metabolic comorbidities, and treatment-related cardiotoxicity, highlighting the need for integrated oncologic–cardiovascular management (5, 6).

This dual disease burden is particularly concerning in India, where cardiovascular disease (CVD) is a leading cause of mortality, with prevalence ranging from 1.6–7.4% in rural areas and 1–13.2% in urban areas, and mortality rising from 15.2% of all deaths in 1990 to 28.1% in 2016, reaching 2.87 million in 2021. Given that CVD is a leading cause of non-cancer mortality in men with prostate cancer, particularly those aged ≥40 years, cardiovascular risk has profound implications for long-term PCa management (7, 8).

Furthermore, ADT, the cornerstone of PCa treatment, is associated with increased cardiovascular risk. GnRH agonists have been associated with elevated risks of diabetes, myocardial infarction, and stroke, with prolonged use (9, 10). In contrast, GnRH antagonists have shown a potentially more favorable cardiovascular safety profile along with comparable efficacy in several studies. However, these findings should be interpreted cautiously, as most evidence is derived from retrospective analyses and meta-analyses, while prospective randomized trials remain limited, underpowered, or prematurely terminated (PRONOUNCE trial), restricting definitive conclusions (11).

In castration-resistant prostate cancer (CRPC), the use of ARPIs including abiraterone, enzalutamide, apalutamide, and darolutamide has significantly improved oncologic outcomes and overall survival. With expanding use across disease stages, including metastatic hormone-sensitive and non-metastatic/metastatic CRPC, ARPIs (abiraterone, enzalutamide, apalutamide, and darolutamide) significantly improve outcomes across the prostate cancer continuum. While they extend survival, ARPIs also carry cardiovascular risks such as hypertension, ischemic heart disease, and arrhythmias (12). With longer survival, Indian patients are now exposed to these therapies for extended periods, potentially increasing the risk of cardiovascular complications. This concern is amplified by the limited applicability of existing cardiovascular safety data, which is predominantly derived from non-Asian populations (13).

Additionally, in India, routine cardiovascular risk assessment and management are not yet fully integrated into prostate cancer care pathways. Oncologists and urologists may lack standardized checklist of risk factors or referral systems to involve cardiologists early in the treatment process, leading to fragmented care and missed opportunities for prevention (12).

These challenges underscore the urgent need for India-specific clinical recommendations that address the intersection of oncology and cardiology in PCa care. A multidisciplinary approach, involving oncologists, urologists, cardiologists, and primary care providers, is essential to reduce cardiovascular morbidity and mortality in PCa patients.

The above conditions underscore the need to build Indian population- based consensus.

## 2 Need for expert consensus

With the rising incidence of CV risk among Indian PCa patients receiving ADT with or without ARPIs, there is a critical need for practical, evidence-based, and India-specific clinical guidance. This expert consensus aims to fill existing gaps in practice by offering actionable recommendations for healthcare providers.

The objectives of this document are to:

• Standardize baseline CV risk assessment before initiating ADT or ARPIs.
• Guide therapy selection with balanced oncologic and cardiometabolic considerations.
• Outline follow-up protocols to prevent, monitor, and manage cardiometabolic complications.

Addressing CV risk across the treatment continuum from screening to long-term care can help improve patient outcomes and reduce non-cancer-related mortality. Given the complex interplay of cancer treatment and cardiovascular health, a multidisciplinary approach is essential. Collaboration among urologists, oncologists, cardiologists, and primary care physicians is key for timely screening, risk stratification, therapy planning, and coordinated follow-up. By incorporating diverse clinical perspectives, this consensus provides relevant and practical guidance tailored to Indian healthcare settings.

## 3 Methodology

The Prostate Cancer Cardiovascular (PCCV) Risk Expert Panel was convened to develop practical, India-specific recommendations for the assessment and management of cardiovascular (CV) risk in patients with prostate cancer (PCa) receiving androgen deprivation therapy (ADT) or androgen receptor pathway inhibitors (ARPIs). The multidisciplinary panel comprised 12 urologists, one medical oncologist, and three cardio-oncologists from across India. Panelists were selected based on their clinical expertise, experience in managing prostate cancer and cardiovascular comorbidities, and their recognition as national experts in India. The panel was further supported by insights from a global cardio-oncology expert.

A modified Delphi methodology was used to develop consensus recommendations. The steering committee prepared a structured questionnaire containing key clinical questions and draft recommendations based on a review of the available evidence and areas of clinical uncertainty. The questionnaire was circulated to all panelists, and the first round of voting was conducted electronically. Voting was anonymous, and responses were collected independently and analyzed in aggregate.

A structured in-person meeting was subsequently held to review the available evidence on the cardiovascular effects of ADT and ARPIs in the context of Indian clinical practice. Panelists discussed the first-round results and provided feedback on the clarity, wording, and applicability of the proposed recommendations. Based on these discussions, statements were refined to improve clarity and clinical relevance.

The consensus process included two voting rounds. Statements that required further clarification or had not achieved sufficient agreement in the first round were revised and re-evaluated through a second round of anonymous voting independent of the sponsor. Final recommendations were based on the voting outcomes, available evidence, and the collective clinical experience of the expert panel.

**Figure 1:**
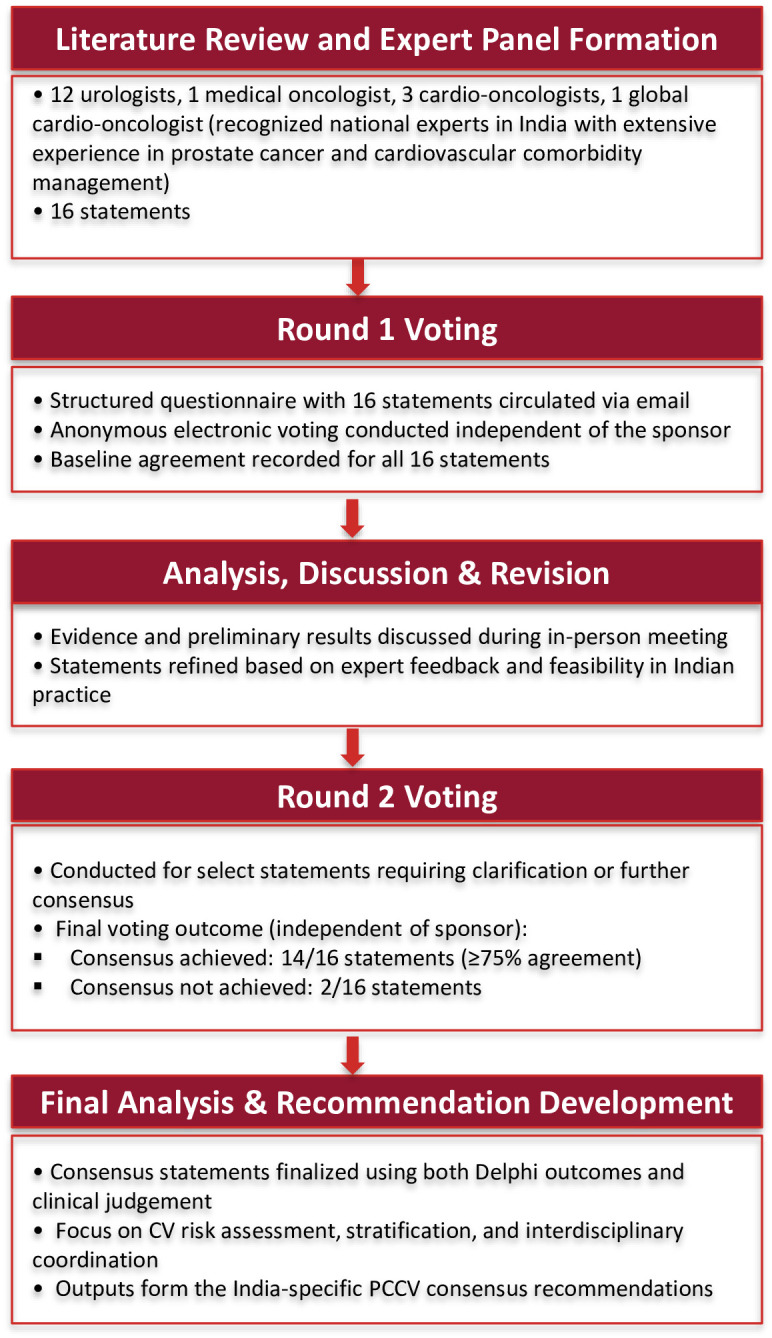
Methodology of the PCCV expert panel consensus process.

Consensus was predefined as agreement by at least 75% of panelists on a given statement. The panel also discussed the practical implementation of CV risk assessment and management in routine clinical practice, leading to recommendations that support risk-based treatment decisions and multidisciplinary collaboration between oncology and cardiology teams (**Figure 1**).

## 4 Expert opinions and panel discussions

### 4.1 Baseline cardiovascular and lifestyle risk assessment

**Statement 1**. A comprehensive baseline assessment of lifestyle risk factors, including smoking, alcohol consumption, substance abuse, physical activity, and diet, is essential before initiating ADT or ARPIs in prostate cancer patients.

Consensus achieved- 90%

Expert opinion:

Indian Experts emphasized the high prevalence of comorbidities among Indian population for diabetes and hypertension, in prostate cancer patients, supporting routine ECG, blood pressure checks, and lipid profiling as part of the baseline assessment. Concerns were raised regarding the applicability of complex global guidelines in Indian clinical practice, highlighting the need for simplified, context-appropriate tools.

The ICMR-INDIAB study reported diabetes and hypertension prevalences of 11.4% and 35.5%, respectively, highlighting the high baseline cardiometabolic risk in the Indian population (14). In a retrospective hospital-based study in prostate cancer patients (n=66), 13.6% had diabetes, while 28.2% had hypertension (4). Lifestyle factors such as smoking and obesity have been found to significantly influence disease progression and overall cardiovascular risk. Smoking is known to affect the androgen milieu, which plays a critical role in the pathogenesis and prognosis of prostate cancer (14, 15).

Additionally, smokers have been shown to have nearly five times higher odds of developing coronary heart disease (CHD) (16). Obesity is another key factor, often exacerbated in men undergoing ADT, further elevating the risk of adverse cardiovascular outcomes (17–19).

Prostate cancer patients receiving ADT commonly have cardiometabolic comorbidities and in the absence of comorbidities, are at increased risk of developing CAD, heart failure, and ischemic stroke compared with those not on ADT. Moreover, in patients receiving ADT, increased variability in visit-to-visit HbA1c levels was observed, which was associated with a significantly elevated risk of major adverse cardiovascular events (MACEs) (20, 21).

Statement 2. A simplified cardiovascular risk assessment checklist including history of cardiovascular disease, smoking, diabetes, hypertension, and substance use should be performed prior to initiating ADT or ARPIs.

Consensus achieved- 100% agreed.

Expert opinion:

The panel supported the use of a simple, structured cardiovascular risk checklist that includes parameters such as smoking status, diabetes, hypertension, history of cardiovascular disease, and substance use. This checklist was considered a practical tool to guide clinical decision-making and ensure timely referrals. Experts emphasized the need to tailor such tools to the Indian clinical setting while maintaining consistency with established cardiovascular guidelines.

**Figure 2:**
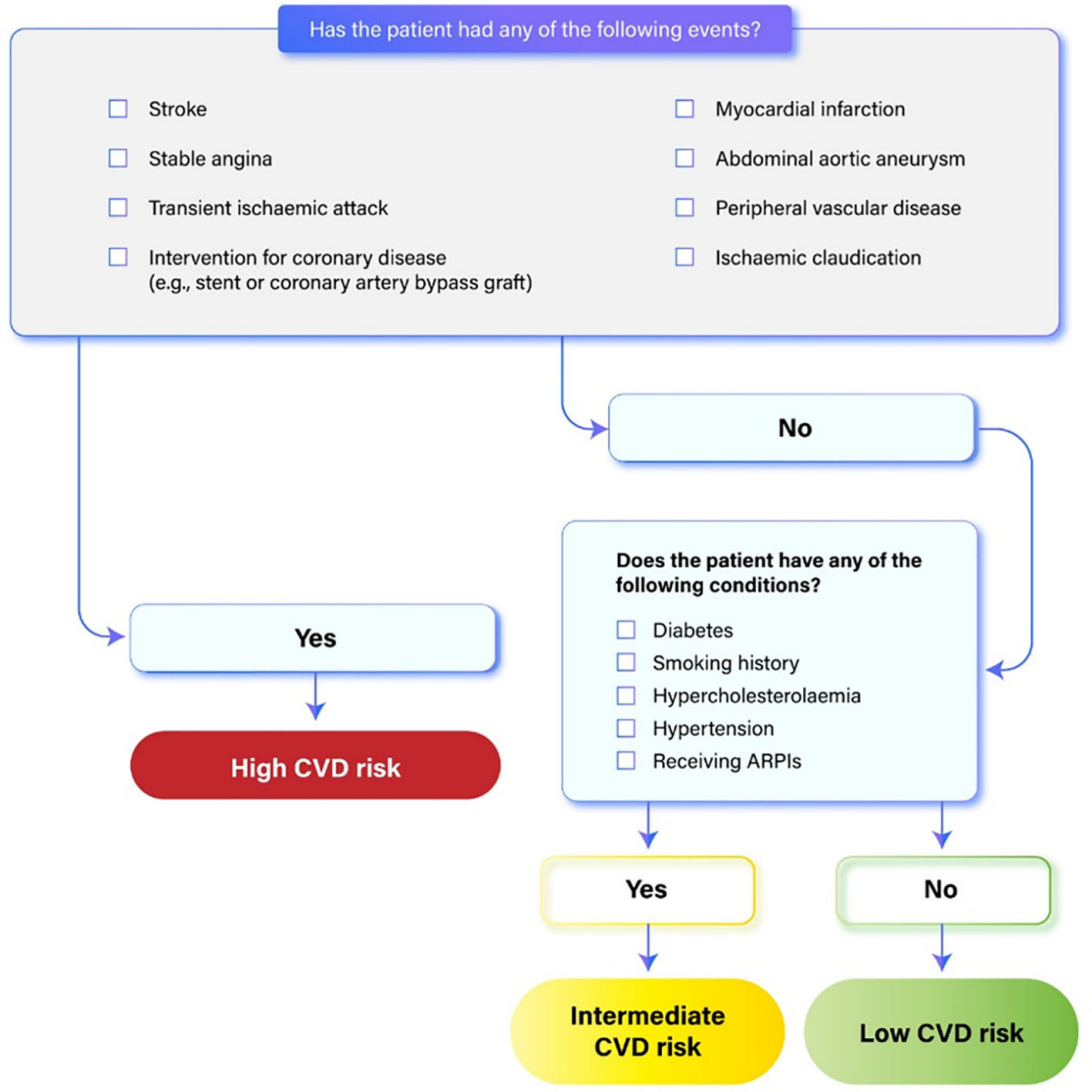
Checklist for CVD risk assessment and stratification. ARPIs, Androgen Receptor Pathway Inhibitors; CVD, Cardiovascular Disease.

Based on the Prostate Cancer Cardiovascular Expert Network, practical strategies to strengthen cardio-oncology care in patients with prostate cancer receiving ADT include streamlined cardiovascular risk assessment, risk-based treatment individualization, multidisciplinary collaboration with clear referral pathways. Clinical algorithms, such as those developed by Merseburger et al, provide structured pathways for decision-making based on CV risk categories. These guidelines underscore the importance of a simplified yet comprehensive CV risk checklist to support early risk identification, personalized treatment planning, and timely specialist involvement (**Figure 2**) (22).

### 4.2 Risk stratification tools and checklists

Statement 3. A baseline electrocardiogram (ECG) is recommended for all patients before starting ADT or ARPIs to screen for underlying cardiac abnormalities.

Consensus achieved - 93% agreed.

Expert opinion:

While recognized as a critical diagnostic tool, experts cautioned against overreliance on AI interpretations in areas with limited cardiology access, particularly tier 2 and 3 cities. Early detection of asymptomatic cardiovascular disease and proactive QT prolongation risk identification were strongly advocated. ECG abnormalities were noted to be influential in treatment decisions, particularly in patients with comorbidities or on polypharmacy.

Baseline ECGs are widely considered a gold standard for assessing cardiac electrical activity and structure (23).

**Figure 3:**
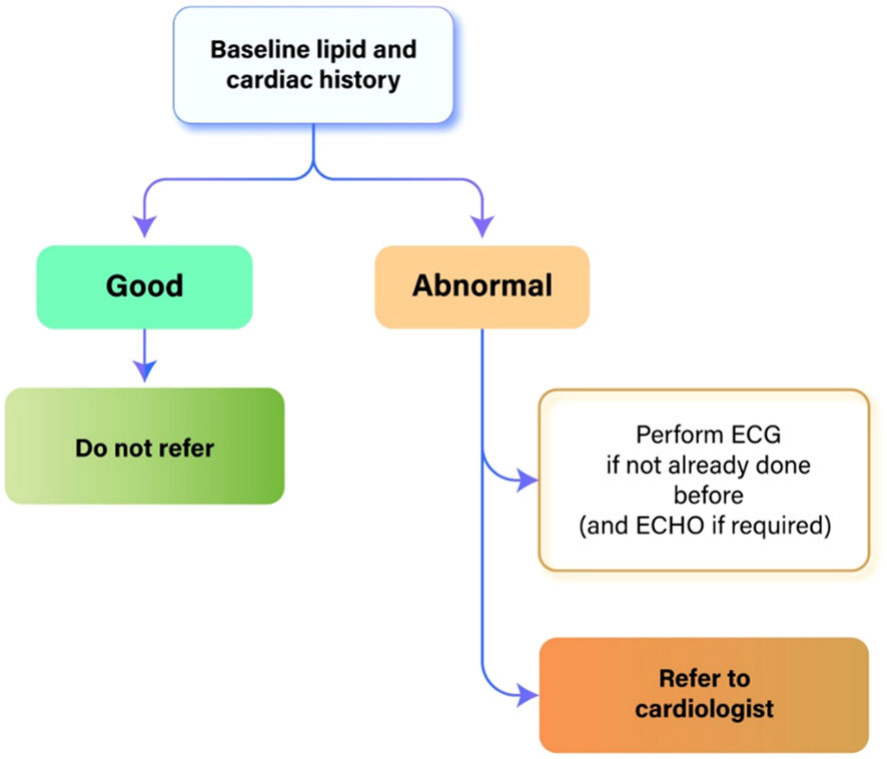
ECG-based cardiac screening before ADT or ARPIs. ECG, Electrocardiography; ECHO, Echocardiography.

Specifically, the presence and number of minor baseline ECG abnormalities have been linked to an increased likelihood of developing significant new ECG abnormalities over time. This evidence supports the role of routine ECG screening in identifying patients who may be at higher risk of adverse cardiovascular outcomes, particularly in the context of prostate cancer therapies known to impact cardiac function (24) (**Figure 3**).

### 4.3 Pre-treatment evaluation protocols

Statement 4. Comprehensive baseline metabolic profiling, including BMI, blood pressure, lipid panel, uric acid, HbA1c, and thyroid function tests, should be mandatory before initiating ADT or ARPIs.

Consensus achieved- 94% agreed.

Expert opinion:

Comprehensive metabolic profiling including HbA1c, lipid levels, thyroid function, and BMI were recommended to uncover latent cardiometabolic risks and personalize treatment.

Both ADT and ARPI are associated with cardiometabolic toxicities, particularly increased risks of hypertension and cardiac events, necessitating careful cardiovascular risk assessment, monitoring, and individualized treatment strategies to balance oncologic benefit with long-term cardiovascular safety (25).

Men, with prostate cancer receiving ADT, have a high prevalence of conventional cardiovascular (CV) risk factors. Nearly all patients (99%) had at least one uncontrolled modifiable CV risk factor, and more than half (51%) had three or more uncontrolled risk factors (25). These included hypertension, diabetes mellitus, dyslipidemia, obesity, and smoking. Importantly, poor control of these risk factors occurred regardless of prior cardiovascular disease or ADT use, emphasizing that CV risk is inherent in this population and not solely therapy-induced (25). To address concern of CV risk, cardiometabolic profiling, including risk factors, comorbidities, and prior heart disease, before starting ADT or ARPIs is necessary (26).

Statement 5. Monthly cardiometabolic monitoring, including blood pressure, BMI, lipid profile, glucose levels, renal and liver function markers, and echocardiography, is advisable after initiating ADT or ARPIs.

Consensus not achieved- Only 50% agreed- No consensus reached.

Expert opinion:

Half the panel opposed routine monthly monitoring. Instead, a tailored strategy based on individual risk profiles was recommended. High-risk patients may benefit from quarterly evaluations, while low-risk individuals might require annual reviews. Experts suggested flexible schedules for different parameters, with frequent checks for BP and glucose, and periodic ECGs and lipid panels based on evolving risk.

According to clinical evidence, long-term ADT, including GnRH agonists, antiandrogens, and orchiectomy, has been shown to elevate the risk of diabetes, particularly with treatments exceeding six months (27). Additionally, ADT use correlates with an increased risk of CVD, including heart failure, and ARPIs may further heighten this risk (28). In a prospective Indian cohort, ADT was associated with a 7.5% incidence of major adverse cardiac events at 1 year and significant increases in Framingham risk score, BMI, and HbA1c compared with controls, thereby necessitating regular monitoring (29).

### 4.4 Monitoring frequency and risk-based follow-up

Statement 6. Risk stratification for cardiovascular disease (low, intermediate, and high) should be integrated into routine assessment of patients undergoing ADT.

Consensus achieved- 90% agreed.

Expert opinion:

The panel strongly agreed that cardiovascular risk should be assessed in a structured way before starting ADT or ARPIs. The APMA risk stratification tool was appreciated for being simple, practical, and well-suited to the local context, offering a more feasible option than complex international guidelines. Experts felt that using a common tool across oncology and urology would bring greater consistency and collaboration. Routine cardiovascular risk assessment was emphasized to guide treatment selection, enable timely cardiology referral, and reduce treatment delays.

**Figure 4:**
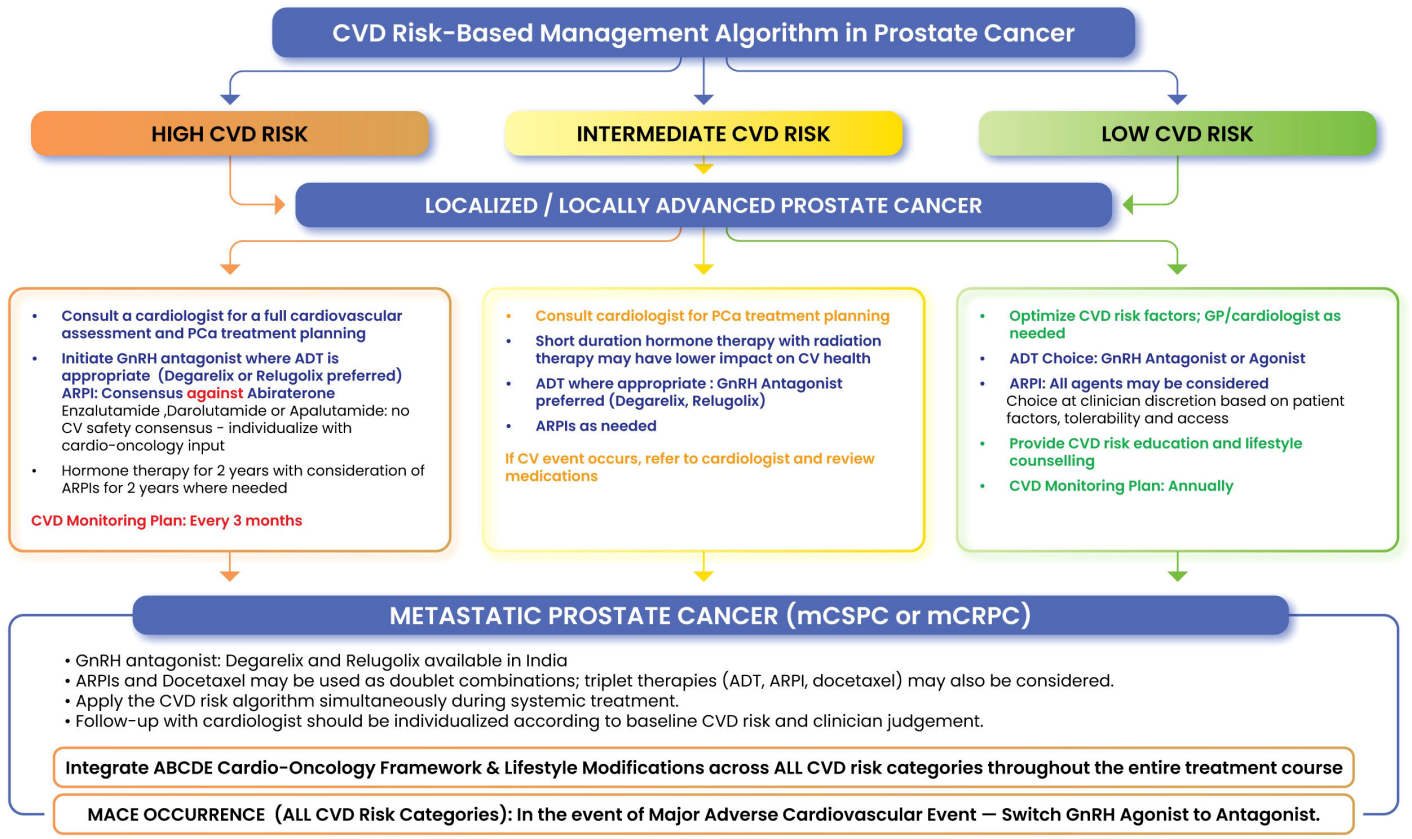
Management pathway for prostate cancer patients on ADT by CVD risk category. ADT, Androgen deprivation therapy; ARPI, Androgen Receptor Pathway Inhibitor; CVD, Cardiovascular disease; GnRH, Gonadotropin-releasing hormone; GP, General Practitioner; mCSPC, metastatic castration-sensitive prostate cancer; mCRPC, metastatic castration-resistant prostate cancer; PCa, Prostate cancer.

ADT should be initiated promptly in patients with low CV risk, while those with intermediate or high CV risk require optimization of modifiable factors before, during, and after therapy. Patients with active cardiac symptoms should be referred to a cardiologist prior to treatment to optimize CV management and minimize treatment interruptions (**Figure 4**) (22).

Statement 7. For patients with intermediate or high cardiovascular risk, referral to a cardiologist or physician should be considered prior to initiating ADT or ARPIs.

Consensus achieved- 100% agreed.

Expert opinion:

Structured CVD risk stratification was endorsed as a key step prior to initiating therapy. A simplified, India-specific tool was favored over complex international models. Experts noted the utility of risk scores in guiding treatment selection (e.g., agonist vs antagonist) and streamlining referrals. Standardized stratification was seen as beneficial for inter-specialty communication and minimizing delays.

Pre-treatment referral to a cardiologist or physician was deemed essential for patients with moderate or high cardiovascular risk. Although logistical challenges in smaller centers were acknowledged, selective referral based on risk levels was considered both feasible and necessary.

Cardiovascular risk stratification remains critical. Treatment may proceed without delay in low-risk patients, whereas those at intermediate or high risk require cardiovascular optimization throughout therapy, with cardiology referral to reduce the likelihood of treatment interruptions from cardiovascular events (22, 30, 31).

In a retrospective monocentric study of prostate cancer patients undergoing ADT, early referral for cardio-oncology evaluation led to the diagnosis of previously unrecognized coronary artery disease in 20.6% of cases, most of whom were asymptomatic. Around 55.9% of patients received therapeutic interventions following referral, with no cardiac deaths during follow-up, underscoring the potential of early cardiology involvement to mitigate cardiovascular risk and improve overall outcomes (32).

Statement 8. In patients with intermediate or high cardiovascular risk, GnRH antagonists are preferred over GnRH agonists to minimize cumulative cardiovascular risk.

Consensus achieved- 90% agreed.

Expert opinion:

Experts agreed that GnRH antagonists should be prioritized for patients with moderate to high cardiovascular risk, given their safer profile, lower rates of adverse events, and suitability for those with diabetes, metabolic syndrome, or existing heart disease.

Importantly, GnRH antagonists are favored over agonists in patients at elevated CV risk, particularly when combined with ARPIs as emerging data suggest they are associated with a lower risk of CV toxicity. Although head-to-head RCT data remain limited, these recommendations aim to minimize cumulative cardiovascular burden (33). Real-world evidence from an observational cohort study showed that, in patients with pre-existing CVD, treatment with antagonists led to a 33% lower risk of MACE (aHR 0.67; 95% CI: 0.46–0.96; p=0.0299) and an 84% reduction in composite CV events (aHR 0.16; 95% CI: 0.05–0.50; p=0.0017) (34). Similarly, the relative risk of cardiac events was 61% lower with antagonists than agonists (RR: 0.39; 95% CI: 0.191–0.799; p=0.01) (33).

The PRONOUNCE trial, the only randomized study specifically designed to evaluate cardiovascular outcomes in patients with prostate cancer and established CVD, reported a numerically higher incidence of MACE with degarelix than with leuprolide; however, the difference was not statistically significant (HR 1.28, 95% CI 0.59–2.79; p=0.53). The findings should be interpreted in the context of early trial termination and lower-than-anticipated event rates (11). In contrast, a real-world evidence meta-analysis by Patel et al. suggests a modestly increased MACE risk with degarelix versus GnRH agonists, particularly in patients with pre-existing CVD (pooled RR: 1.31; 95% CI: 1.11–1.56), although residual confounding and channeling bias may explain these findings, warranting further robust studies (35).

### 4.5 Treatment choices based on CV risk

Statement 9. In patients with preexisting cardiovascular disease, GnRH antagonists are generally favored over GnRH agonists due to a more favorable cardiovascular safety profile.

Consensus achieved- 90% agreed.

Expert opinion:

Antagonists were preferred due to their favorable cardiovascular safety profiles, especially for patients with diabetes, metabolic syndrome, or existing CVD. Clinical experience supported this trend, and experts pointed to growing real-world data favoring antagonists in higher-risk groups, despite evolving evidence for ARPIs, alongside routine monitoring to support ongoing management.

Based on retrospective data, GnRH antagonists have demonstrated favorable cardiovascular safety profile compared to GnRH agonists, especially in patients with pre-existing CVD (36). Evidence shows that in patients receiving ADT for over six months, GnRH antagonists were associated with a 54% lower risk of major adverse cardiovascular events (MACE), owing to their rapid and sustained testosterone suppression. Unlike agonists, antagonists do not activate T lymphocytes, thereby helping maintain plaque stability (33, 37).

Studies have shown that patients treated with GnRH antagonists had a 61% lower relative risk of cardiac events compared to those on GnRH agonists (RR: 0.39; 95% CI: 0.191–0.799; p=0.01) (22). The 2022 ESC guidelines recommend the use of GnRH antagonists in patients with symptomatic coronary artery disease who require ADT. Additionally, antagonists have shown lower cardiovascular mortality and thrombotic risk compared to surgical castration, with a reduced incidence of acute myocardial infarction (AMI) in advanced cancer stages (38).

However, data from systematic reviews and comparative analyses have reported comparable risks of MACE, stroke, myocardial infarction, arrhythmia, and all-cause mortality between degarelix and GnRH agonists, although degarelix may confer a lower risk of heart failure. These inconsistent findings highlight the need for adequately powered prospective randomized trials to better define the cardiovascular effects of different ADT modalities (39).

The PRONOUNCE trial, the only randomized study specifically designed to evaluate cardiovascular outcomes in patients with prostate cancer and established CVD, reported a numerically higher incidence of MACE with degarelix than with leuprolide; however, the difference was not statistically significant (HR 1.28, 95% CI 0.59–2.79; p=0.53). The findings should be interpreted in the context of early trial termination and lower-than-anticipated event rates (11). Real-world studies analyses by Patel et al., report increased cardiovascular risk with degarelix compared with GnRH agonists such as leuprolide, likely reflecting residual confounding and channeling bias (35).

Statement 10. Patients receiving ADT should have follow-up cardiology assessments at least every 6 months or annually to monitor cardiac status.

Consensus achieved- 90% agreed.

Expert opinion:

Experts recommended annual follow-ups for low-risk patients and 6-monthly reviews for high-risk individuals. Dynamic monitoring based on evolving clinical parameters such as BP, glucose levels, and QTc interval was advised to guide timely intervention and treatment modification.

In patients receiving ADT, cardiovascular (CV) follow-up is essential, with ESC guidelines suggesting a tailored approach based on individual risk (22). An annual CV risk assessment including ECG, natriuretic peptides (NP), and risk factor evaluation is advised for cancer survivors exposed to potentially cardiotoxic therapies (40).

During the first year of ADT, it is recommended that metabolic assessments be conducted at 6 months and again at 12 months, followed by individualized testing frequency based on the patient’s evolving risk profile and clinical status. This stratified approach ensures timely identification and management of cardiometabolic complications during ADT (41).

### 4.6 Role of lifestyle interventions and multidisciplinary management

Statement 11. Lifestyle modification counselling, including diet, physical activity, smoking cessation, and stress management, should be incorporated into follow-up visits to support cardiovascular risk management.

Consensus achieved- 100% agreed.

Expert opinion:

Lifestyle interventions such as smoking cessation, exercise, diet changes, and stress management were strongly endorsed by experts. A comprehensive approach including referrals to physiotherapists, dietary counselling, and patient-specific guidance was recommended. Pharmacologic interventions such as GLP-1 receptor agonists and SGLT2 inhibitors were noted as valuable adjuncts in high-risk patients.

Initiating exercise at the onset of ADT in men with prostate cancer significantly preserves muscle strength and physical function. In a randomized trial involving 104 men, those in the immediate exercise group showed greater improvements at 6 months in leg press strength (+19.9 kg), seated row (+5.6 kg), and chest press (+4.3 kg) compared to delayed exercise (P < 0.001). Physical function also improved significantly, with faster times in the 6-m fast walk (−0.2 s), 400-m walk (−9.7 s), stair climb (−0.4 s), and chair rise (−1.0 s) (42). In prostate cancer patients receiving ADT, 20 weeks of resistance exercise training significantly improved muscle mass and strength and helped preserve aerobic capacity, counteracting the adverse effects of ADT (43).

In prostate cancer patients initiating ADT, immediate commencement of a 6-month supervised resistance/aerobic/impact exercise program preserved health-related quality of life (HRQoL), particularly in physical functioning and vitality domains, compared to delayed exercise. At 6 months, the immediate exercise group maintained HRQoL, while the delayed group showed declines (physical component summary score interaction: *p* = 0.005; physical functioning: *p* = 0.045). Initiating exercise later reversed some of these effects by 12 months (44). These findings further support the incorporation of structured supportive care and lifestyle-based intervention programs during ADT to improve long-term functional and cardiometabolic outcomes. Patient-support initiatives such as the Feel+ programme endorsed by the European Prostate Cancer Coalition have also emerged as supplementary tools aimed at encouraging physical activity, dietary awareness, and holistic wellbeing in patients receiving hormonal therapy (45).

### 4.7 Integration of ABCDE cardio-oncology framework

Statement 12. The ABCDE Cardio-Oncology framework should be integrated into the routine management of patients receiving ADT or ARPIs.

Consensus achieved- 90% agreed.

Expert opinion:

The ABCDE (A – Awareness and Aspirin, B – Blood Pressure, C – Cholesterol/Cigarettes, D – Diet/Diabetes, E – Exercise) framework was endorsed, with modifications to suit local dietary and cost constraints. Imaging frequency (e.g., ECHO) and regional preferences were considered important for effective integration. Experts emphasized the importance of adopting a balanced diet aligned with local food availability and cultural habits. Both aerobic and strength-training exercises were highlighted to maintain cardiometabolic health.

The ABCDE Cardio-Oncology framework, endorsed by the NCCN Guidelines for Survivorship, offers a structured approach to addressing CVD risk factors in cancer survivors (46). Specifically adapted for prostate cancer patients on ADT, the ABCDE paradigm supports the prevention and management of CVD through comprehensive measures such as cardiovascular risk assessment, blood pressure control, lifestyle modifications, and monitoring, and has demonstrated utility in cardio-oncology clinics for routine use (47, 48).

Statement 13. An integrated care model, involving cardiologists, endocrinologists, and primary care providers, is essential for managing cardiovascular complications during ADT or ARPIs.

Consensus achieved- 75% agreed.

Expert opinion:

A multidisciplinary team approach involving urologists, oncologists, cardiologists, endocrinologists, and general physicians was strongly supported. Experts stressed that coordinated care improves medication safety, monitoring accuracy (e.g., ECG/QTc), and patient education, ultimately enhancing adherence and outcomes.

An integrated care model leveraging multidisciplinary teams as above do enhance the management of complex malignancies such as prostate cancer (49).

This approach is essential for personalized, comprehensive care and optimized outcomes.

Such models enable the combined expertise of various specialists to address the multifaceted needs of patients undergoing ADT or ARPIs (50).

### 4.8 Safety considerations in drug selection

Statement 14. When selecting a GnRH antagonist, patient compliance, QTc interval considerations and LV function were identified as key factors influencing treatment choice.

Consensus achieved- Patient compliance- 100%, QTc interval- 90%, LV function-80%

Expert opinion:

Therapy selection should account for QTc risk and drug interactions. Experts highlighted the importance of reviewing concurrent medications that may prolong QT intervals. Pre-treatment ECGs and a thorough medication review were recommended, especially in patients with known LV dysfunction or on polypharmacy.

When selecting a GnRH antagonist, assessment of QTc risk represents a critical component of clinical decision-making. Identification of pre-existing ECG QTc prolongation is recommended, which is a surrogate marker for treatment induced arrhythmias. In selected patients, regular prospective monitoring with ECG enhances patient safety (51).

Specifically, a randomized crossover study demonstrated that degarelix does not intrinsically prolong the QT interval or affect cardiac repolarization, even at supratherapeutic concentrations, suggesting an absence of direct QT liability associated with degarelix (52).

The influence of drug–drug interactions on QTc prolongation warrants particular attention in patients undergoing ADT, where polypharmacy is common. Both pharmacokinetic and pharmacodynamic interactions can exacerbate cardiac risk by altering ion channel activity or interfering with metabolic pathways, leading to cumulative QTc effects. Consequently, a comprehensive review of concurrent medications prior to initiating a GnRH antagonist is essential to minimize the potential for additive proarrhythmic effects (53).

Statement 15. In patients presenting with red flag signs such as unstable cardiovascular events, ECG QTc prolongation, or coronary symptoms treatment modification or withdrawal should be considered.

Consensus achieved- 81% agreed.

Expert opinion:

Panelists advised against discontinuing ADT abruptly. Instead, switching from agonist to antagonist and addressing the cardiovascular issue with evidence-based treatment was preferred. Lifestyle adjustments and cardioprotective agents like GLP-1 agonists or SGLT2 inhibitors were suggested. Early cardiology involvement was emphasized.

GLP-1 receptor agonists and SGLT2 inhibitors have been associated with reduced prostate cancer risk in men with diabetes, with GLP-1RA showing an OR of 0.53 and SGLT2 inhibitors a 23% lower incidence (HR 0.77) (54, 55). Evidence from pooled analyses and randomized trials indicates that GnRH antagonists, such as degarelix and relugolix, significantly reduce cardiovascular events compared to agonists, with hazard ratios of 0.44 and 0.46, respectively, and up to a 54% reduction in major cardiovascular events among patients with pre-existing cardiovascular disease (25). Accordingly, switching from a GnRH agonist to an antagonist is preferred over abrupt discontinuation of therapy to maintain oncologic efficacy while mitigating cardiovascular risk (**Table 1**) (11, 25, 37, 56, 57).

**Table 1:** Comparative overview of GnRH antagonists vs. GnRH agonists in androgen deprivation therapy.

Parameter	GnRH antagonists (degarelix, relugolix)	GnRH agonists (leuprolide, goserelin)
Cardiovascular Profile	Associated with lower incidence of major adverse cardiovascular events (MACE) in pooled analyses and the HERO trial (Relugolix: HR 0.46, 95% CI 0.24–0.88) (37)	PRONOUNCE reported a numerically higher, but non-significant, incidence of MACE with degarelix versus leuprolide (HR 1.28, 95% CI 0.59–2.79; p=0.53), while Patel et al. observed a modest increase in MACE risk with degarelix compared with GnRH agonists (pooled RR 1.31; 95% CI 1.11–1.56). These findings should be interpreted in light of the limitations of both studies (11, 35)
Use in Patients with Established Cardiovascular Disease	Preferred in men with pre-existing cardiovascular disease due to favorable CV risk profile and absence of testosterone surge (25, 37)	May exacerbate cardiovascular risk in susceptible individuals due to transient hormonal flare and plaque destabilization (56, 57)
Reversibility and Compliance	Relugolix (oral) provides rapid reversibility and improved convenience/adherence (37)	Depot formulations require parenteral administration and result in prolonged hormonal suppression (56)

However, the evidence remains heterogeneous. The PRONOUNCE trial, the only randomized study specifically designed to evaluate cardiovascular outcomes in patients with prostate cancer and established CVD, reported a numerically higher incidence of MACE with degarelix than with leuprolide; however, the difference was not statistically significant (HR 1.28, 95% CI 0.59–2.79; p=0.53). The findings should be interpreted in the context of early trial termination and lower-than-anticipated event rates (11). Real-world studies, including those by Patel et al., report increased risk with GnRH antagonists, likely reflecting residual confounding and channeling bias (35). Overall, these findings warrant cautious interpretation and individualized decision-making.

Statement 16. Guideline-directed medical therapy (GDMT) should be implemented in all patients undergoing ADT or ARPIs, especially those with cardiovascular risk.

Consensus not achieved- 20% agreed – No consensus reached.

Expert opinion:

The panel emphasized that guideline-directed medical therapy (GDMT) should not be performed in all patients. GDMT in heart failure is supported by four pillar therapies, Angiotensin Receptor Neprilysin Inhibitor, beta-blockers, SGLT2 inhibitors, and Mineralocorticoid Receptor Antagonists. Its use should be restricted to those with left ventricular (LV) dysfunction or heart failure (including subclinical cases), where continuation of GDMT is essential even when cardiac function appears to have recovered. In contrast, patients with preserved LV function or those with stable coronary artery disease and isolated metabolic or lipid abnormalities may not require GDMT universally. In such cases, therapy should be individualized based on clinical response and follow-up.

Clinical evidence suggests early initiation of GDMT in HFrEF reduces mortality and hospitalizations, while underuse or underdose worsens outcomes. HFrEF predominates in India, presents earlier, and carries high mortality, necessitating early comprehensive GDMT to reduce disease progression and lifetime burden. GDMT underuse in India reflects therapeutic inertia, safety concerns, limited guideline applicability, and barriers related to drug availability and affordability (58).Withdrawal of GDMT in patients with improving chemotherapy-related LV dysfunction (CTRCD) is linked to adverse outcomes. A study demonstrated that stopping GDMT in these patients resulted in deterioration of left ventricular function and poorer clinical outcomes (59).

**Table 2** summarizes the outcomes of the consensus.

**Table 2:** Summary of consensus outcomes.

Statement no.	Statement	% Agreement
	Baseline cardiovascular and lifestyle risk assessment	
1	A comprehensive baseline assessment of lifestyle risk factors, including smoking, alcohol consumption, substance abuse, physical activity, and diet, is essential before initiating ADT or ARPIs in prostate cancer patients.	Consensus reached (90)
2	A simplified cardiovascular risk assessment checklist including history of cardiovascular disease, smoking, diabetes, hypertension, and substance use should be performed prior to initiating ADT or ARPIs.	Consensus reached (100)
	Risk stratification tools and checklists	
3	A baseline electrocardiogram (ECG) is recommended for all patients before starting ADT or ARPIs to screen for underlying cardiac abnormalities.	Consensus reached (93)
	Pre-treatment evaluation protocols	
4	Comprehensive baseline metabolic profiling, including BMI, blood pressure, lipid panel, uric acid, HbA1c, and thyroid function tests, should be mandatory before initiating ADT or ARPIs.	Consensus reached (94)
5	Monthly cardiometabolic monitoring, including blood pressure, BMI, lipid profile, glucose levels, renal and liver function markers, and echocardiography, is advisable after initiating ADT or ARPIs.	Consensus not reached (50)
	Monitoring frequency and risk-based follow-up	
6	Risk stratification for cardiovascular disease (low, intermediate, and high) should be integrated into routine assessment of patients undergoing ADT.	Consensus reached (90)
7	For patients with intermediate or high cardiovascular risk, referral to a cardiologist or physician should be considered prior to initiating ADT or ARPIs.	Consensus reached (100)
8	In patients with intermediate or high cardiovascular risk, GnRH antagonists are preferred over GnRH agonists to minimize cumulative cardiovascular risk.	Consensus reached (90)
	Treatment choices based on CV risk	
9	In patients with preexisting cardiovascular disease, GnRH antagonists are generally favored over GnRH agonists due to a more favorable cardiovascular safety profile.	Consensus reached (90)
10	Patients receiving ADT should have follow-up cardiology assessments at least every 6 months or annually to monitor cardiac status.	Consensus reached (90)
	Role of lifestyle interventions and multidisciplinary management	
11	Lifestyle modification counselling, including diet, physical activity, smoking cessation, and stress management, should be incorporated into follow-up visits to support cardiovascular risk management.	Consensus reached (100)
	Integration of ABCDE cardio-oncology framework	
12	The ABCDE Cardio-Oncology framework should be integrated into the routine management of patients receiving ADT or ARPIs.	Consensus reached (90)
13	An integrated care model, involving cardiologists, endocrinologists, and primary care providers, is essential for managing cardiovascular complications during ADT or ARPIs.	Consensus reached (75)
	Safety considerations in drug selection	
14	When selecting a GnRH antagonist, patient compliance, QTc interval considerations and LV function were identified as key factors influencing treatment choice.	Consensus reached (Patient compliance- 100, QTc interval- 90, LV function-80)
15	In patients presenting with red flag signs such as unstable cardiovascular events, ECG QT prolongation, or coronary symptoms treatment modification or withdrawal should be considered.	Consensus reached (81)
16	Guideline-directed medical therapy (GDMT) should be implemented in all patients undergoing ADT or ARPIs, especially those with cardiovascular risk.	Consensus not reached (20)

## 5 Future directions and limitations

The recommendations presented herein are based on the best available evidence, complemented by expert consensus, with a substantial proportion of the current evidence base derived from retrospective studies. While these data provide important clinical insights, there remains a need for well-designed prospective studies to further strengthen the evidence base and enable refinement and validation of risk assessment strategies and management approaches in this population.

An important futuristic focus is the development and validation of standardized cardiovascular risk assessment models tailored to patients with prostate cancer. In the interim, a pragmatic approach may involve adapting existing cardiology risk scores by incorporating cancer- and treatment-specific modifiers, such as androgen deprivation therapy exposure, treatment duration, metabolic alterations, and systemic inflammatory burden. Such adaptations have the potential to improve risk stratification and inform clinical decision-making, pending prospective validation to ensure their accuracy and generalizability.

Finally, translating these recommendations into routine practice requires addressing real-world implementation barriers, particularly in resource-limited settings. Limited access to cardio-oncology expertise, cost constraints, and delays in referral pathways remain key challenges. In such contexts, where specialist availability is often scarce, strengthening physician education and awareness is essential to support early cardiovascular risk identification and initial management at the primary care or oncology level. Scalable strategies, including simplified screening pathways and tiered care models, with selective referral of high-risk patients, may enhance feasibility and facilitate broader integration of multidisciplinary care. Future research should also evaluate the effectiveness of these implementation approaches across diverse healthcare settings.

## 6 Conclusion

Due to ageing being a shared predisposing factor for prostate cancer and cardiovascular diseases, the Indian population in the current era is increasingly susceptible to medical conditions such as diabetes mellitus, obesity, and cardiovascular diseases. As the use of ADT and ARPIs continues to expand in the management of prostate cancer, the associated cardiovascular risks demand proactive, evidence-aligned mitigation strategies.

This expert consensus underscores the critical need for baseline cardiovascular risk assessment, routine cardiometabolic monitoring, and individualized treatment decisions particularly in patients with pre-existing cardiovascular disease or elevated risk. GnRH antagonists may offer a safer profile in such patients, and referral to cardiology should be considered for those at intermediate or high cardiovascular risk.

Individualized GDMT should be selectively applied under cardiologist supervision, ensuring personalized care for prostate cancer patients, that balances oncologic efficacy with cardiovascular safety. A multidisciplinary, integrated care model is essential to optimize long-term outcomes and quality of life for this growing patient population.
